# Supplementary material for: Are carbon emissions trading and green financial instruments synergistic? -Comprehensive quantitative research based on content analysis
Source: PLoS One. 2024 Mar 7;19(3):e0298601. doi: 10.1371/journal.pone.0298601 (PMC10919601; doi:10.1371/journal.pone.0298601)
Supplement: S1 Data — (ZIP) [file pone.0298601.s001.zip › Data/地级市Z_F工作报告.zip!/╕╩╦α/╛╞╚¬╩╨/└┤╫╘╣1⁄2╓┌║┼í╛┬φ┐╦╩2╛▌═°í┐_2017.docx]

各位代表:

　　现在，我代表市人民政府向大会作工作报告，请予审议，并请各位政协委员和列席人员提出意见。

　　2016年及本届政府工作回顾

　　2016年，是全面建成小康社会决胜阶段的开局之年，也是推进供给侧结构性改革的攻坚之年。面对经济下行压力持续、改革任务艰巨繁重、外部环境很不宽松的多重困难和严峻挑战，在市委的坚强领导下，我们全面贯彻党的十八大和十八届三中、四中、五中、六中全会精神，以邓小平理论、“三个代表”重要思想和科学发展观为指导，深入贯彻习近平总书记系列重要讲话精神，协调推进“五位一体”总体布局和“四个全面”战略布局，认真落实省委、省政府决策部署和市委“五做五加强”工作思路，圆满完成了市三届人大六次会议确定的目标任务，实现了“十三五”规划的良好开局。预计，全市完成生产总值600亿元，增长7.5%；固定资产投资1215亿元，增长10%；社会消费品零售总额192.5亿元，增长9%；财政总收入108.7亿元，增长8%，公共财政预算收入37.3亿元，增长8%；城镇居民人均可支配收入30016元，增长8%；农村居民人均可支配收入14963元，增长10%；脱贫攻坚目标提前实现，贫困发生率下降至0.36%。政府承诺的10件惠民实事，除公益性蔬菜市场正在建设外，其余9件全面完成。一年来，我们的主要工作成效是:

　　1.首届敦煌文博会成功举办。抢抓丝绸之路国际文化博览会在敦煌永久举办的重大机遇，在省委、省政府和市委的正确领导下，加强组织协调，明确责任分工，仅用9个月的时间，建成了国际会展中心、敦煌大剧院、国际酒店等核心场馆，完成了敦煌机场扩建、市容美化亮化、宾馆饭店改造、旅游景区提升等配套工程，综合保障、环境整治、群防群治、网络管理等工作保障有力，确保了来自全球85个国家、5个国际组织的1700多位中外嘉宾参与的高峰会议、文化年展、文艺演出等活动圆满成功举办，达到了推动“一带一路”沿线国家和地区文化融合、思想融汇、感情融通的目的，唱响了文博盛会国际品牌。

　　2.固定资产投资平稳增长。坚持把抓项目、促投资作为稳增长调结构的重要抓手，层层靠实责任，实行项目“挂图作战”和督查通报制度，开工建设重大项目2594个，其中亿元以上项目244个，争取国投省扶资金52.2亿元，增长25.6%。柳敦公路和白明高速、额哈铁路、敦格铁路酒泉段建成通车，敦当高速开工建设，肃航公路完成路基工程，百立通用机场游客接待中心建成，酒额铁路、敦煌客运专线等项目前期工作进展顺利。灌区续建配套、中小河流治理、湿地保护等农林水利项目完成年度任务。结合编制“十三五”规划，认真研究国家产业政策和投资导向，狠抓项目论证和争取衔接，谋划生成项目1474个，获准进入国家和省上PPP项目库18个。千方百计扩大招商引资规模，着力推进产业链招商，成功举办第七届新博会，开工各类招商项目1056个，引进到位资金714亿元。

　　3.产业转型迈出坚实步伐。深入推进供给侧结构性改革，注重在优化结构、延伸链条、推进转型上下功夫，三次产业结构调整为14.4:37.1:48.5。创新农业经营模式，强化政府引导、企业带动和科技支撑，新增日光温室5377亩、钢架大棚1.9万亩，定植特色林果14.9万亩，新增3000元以上高效田8万亩，新建扩建规模养殖场125个，新增肉羊33万只；积极推进农业标准化生产和规模化经营，农村土地规模经营达到56万亩，新认定农民专业合作示范社71家，新建改建产业化龙头项目25个，农产品加工增值率达到64%；全面落实农产品质量检测和动物疫情防控措施，农产品抽检合格率达到99%。认真落实“止滑稳增”二十条措施，着力解决企业生产、资金融通、产品开发、市场营销等瓶颈问题，工业增加值增长4%。新能源二期项目稳步推进，500万千瓦风电项目可在年内批复核准，400万千瓦调峰火电具备开工条件，建成光热发电装机20.2兆瓦，实现了零的突破。大唐八0三电厂66万千瓦热电联产项目并网发电；酒泉至湖南±800千伏特高压直流输电工程提前完成年度建设任务，阿克塞330千伏输变电工程并网运行，我市首个售电侧改革专项试点园区获批。金塔金源、玉门浩海、瓜州鑫源等煤化工项目和万里鑫农可降解农膜、西域阳光甜叶菊加工等新型工业项目建成投产。推进现代服务业综合改革试点，巨龙物流港、龙德盛农产品交易市场建成运营，万达商业广场、酒泉种子产业园、金塔航天商业街等大型商贸项目加快推进，酒泉经开区创建为省级生产性服务业示范区，酒嘉物流中心和大禹节水列为省级生产性服务业示范企业。金塔、玉门、瓜州、敦煌、阿克塞5个电商服务中心开业运营，全市建成乡村电商服务站点38个，网上交易额达到48亿元。大型室内情景剧《又见敦煌》隆重上演，成功举办第二届酒泉国际戈壁超级马拉松、第十一届“玄奘之路”戈壁挑战赛、第七届金塔胡杨文化旅游节等17项重大赛事节会，旅游接待人数和旅游收入双双增长25%以上。

　　4.城乡建设展现新的面貌。酒泉市历史文化名城保护规划、城市风貌规划、交通专项规划编制和地下管线普查基本完成，酒泉城区南滨河路、风光大道等11条道路和金河大桥建成通车，第二水厂迁建工程投入运行，新城区二期热源厂及6座换热站建成供暖，新增供热面积200万平方米。全国园林城市、国家卫生城市和全国文明城市创建深入推进，锦玉公园、中医药养生园、滨河路绿色景观廊道建成向市民开放，新增绿化面积188万平方米，市容市貌、交通秩序、市场环境整治取得明显成效。积极推进棚户区改造工程，全市建成保障性住房2.68万套。加快推进城乡一体化，全市新增农村安全用水居民1.6万人，新续建楼居式社区化中心村12个，建成美丽乡村示范点36个，敦煌市、玉门市和航天镇、总寨镇“多规合一”试点达到省定考核目标。新建提升农村公路960公里，实现了乡乡通油路。全面放开城乡落户限制，城镇化率比上年提高1.6个百分点。着力改善城乡生态环境，全市完成人工造林19.5万亩，封滩育林6万亩，义务植树497万株；实施减排及污染防治项目54个，万元GDP能耗下降2.75%，单位工业增加值用水量控制在省定目标以内，截至10月底，市区空气质量优良天数达到245天。

　　5.移民乡村实现整体脱贫。认真贯彻落实中央和省委、省政府精准扶贫政策，强化干部帮扶责任，对接出台配套政策，实行跟踪服务、动态管理、限期脱贫、巩固成果等措施，全市落实帮扶资金8.2亿元，实施基础设施建设项目100余项。强化扶智工程，培训输转劳务1.1万人。坚持富民产业培育，移民乡村新增枸杞、甘草等高效田9.1万亩，新建日光温室和钢架大棚1318座，新增暖棚圈舍1032座，调引基础母羊3.4万只，移民乡村高效特色产业比重达71%。建设光伏扶贫项目25.7兆瓦，贫困乡村电商扶贫站点实现全覆盖，移民人均纯收入预计达到8000元以上，减贫人口2.42万人。

　　6.改革创新持续深入推进。落实非公经济发展激励措施，制定出台“双创”推进方案和众创空间管理办法，降低创业门槛，放宽市场准入，大力扶持“互联网+”新技术和众创、众包、众扶、众筹新模式。全面推开营改增税制改革和“五证合一、一照一码”登记制度，发放创业担保贷款6034万元，扶持创业实体955个，新增各类市场主体1.34万户，实现非公经济增加值315.6亿元，增长12%，占GDP的比重提高到52.6%。强化科技创新引领，引导全社会加大科技研发经费投入，大力开展企业技术创新活动，加快先进技术引进消化吸收和再创新，培育发展高新技术企业和创新型企业，新增授权专利520件，有效发明专利达到126件。深化财政和投融资体制改革，盘活财政存量资金2.37亿元，政府性债务管理不断规范，兴业银行、浙商银行、银河证券入驻筹建，招商证券酒泉营业部组建成立，8家企业在股权交易中心挂牌，直接融资5.2亿元，增长7.8倍。稳妥推进涉农领域改革，集体土地所有权、建设用地使用权和宅基地使用权确权登记覆盖面达100%。农业设施产权登记颁证试点顺利启动，土地流转面积达到61万亩，金塔、敦煌分别被确定为全省开展农村承包经营权抵押贷款、农村土地承包经营权入股试点县。公车改革全面实施，司勤人员安置、公务用车划转、管理平台建设正在有序推进，公车运行维护费下降7.5%。

　　7.民生保障水平不断提高。认真落实就业扶持政策，争取就业补助资金1.06亿元，购买公益性岗位1225个，帮助2337名困难人员实现就业，选拔扶持1353名高校毕业生到基层和企业服务，新增城镇就业3.5万人。继续提高社会保障待遇水平，企业退休人员养老金提高8.1%，城乡居民医疗保险筹资标准提高7%，城乡低保标准提高10%，五保集中供养标准提高63%，城乡居民基本社会保险参保率保持在98%以上，发放城乡低保、五保供养等保障救助资金4.2亿元。加快推进健康养老服务工程，7县市区老年养护中心开工建设。全面落实学前教育幼儿保教费补助政策，发放保教费补助金和贫困家庭救助金1.3亿元。实施教育改薄工程205项，新建维修校舍11万平方米，新增幼儿园12所，酒泉北苑学校、工贸中专新校区、实验幼儿园等学校建成使用。深化医疗卫生体制改革，我市被国家确定为第四批公立医院改革试点城市，10家县级公立医院实行药品、医用耗材零差率销售，近400名医师赴基层开展多点执业，县域内就诊人数比例达到85%。文化遗产“历史再现”工程加速推进，新增酒泉民俗、玄奘取经等博物馆6个。新建“乡村舞台”75个，玉门市、阿克塞县全民健身中心和肃北县体育馆建成，成功举办第四届酒泉市运动会、第四届酒泉华夏·文化艺术节等群众性文体活动。强化安全生产监管，狠抓重点行业和领域安全综合治理，安全生产四项指标全面下降。食品药品网格化监管覆盖面达100%。加大矛盾纠纷排查调处力度，完善社会治安防控体系，严厉打击各类违法犯罪活动，维护了社会大局和谐稳定。军民共建和国防动员深入推进。办理人大代表意见建议和政协委员提案387件，办复率达到100%。统计、人防、边防、供销、地震、气象、妇女、儿童、残疾人、台侨、民族、宗教、档案、史志、外事等工作取得新的成绩。

　　各位代表，本届政府任期已满。五年来，我们主动适应引领新常态，着力化解经济下行等不利因素影响，团结带领广大干部群众奋力拼搏、艰苦创业，圆满完成了“十二五”规划和市三届人大一次会议确定的目标任务，经济社会发展取得新的巨大成就。

　　——过去的五年，我们抢抓政策叠加机遇，着力强化项目支撑，基础设施条件明显改善，经济发展后劲显著增强。积极融入“丝绸之路经济带”发展战略，着力放大丝绸之路(敦煌)国际文化博览会、新能源产业博览会、国家级经济技术开发区和现代农业示范园区等节会和平台效应，大力实施“3341”项目工程，持续推进重大项目建设，全市开工建设亿元以上重大项目1372个，累计完成固定资产投资4286亿元，是“十一五”的3.8倍，一大批农林水利、交通通信、电力能源、生态民生等基础设施项目建成投运，有力支撑了经济社会持续健康发展。全市生产总值年均增长8%，人均GDP突破8000美元，第三产业比重提高10.4个百分点。财政总收入实现翻番，跨上百亿元台阶，年均增长12.6%，高于经济增速4.6个百分点。城乡居民人均可支配收入年均增长12.9%，稳步进入中等收入行列。

　　——过去的五年，我们全面推进各项改革，着力激发市场活力，创业创新热潮竞相迸发，市场主体内生动力增强。大力推进简政放权改革，先后取消、调整和下放行政审批事项9批402项，放宽投资项目审批、核准、备案权限，理顺市县政府权责清单，加大政务公开力度，“三张清单一张网”全面推行。五年各类市场主体增长79.2%，设立院士工作站5个、企业工程技术研究中心67个，培育形成众创空间30个，建成国家级高新技术企业23户、省级技术创新示范企业3户，战略性新兴产业增加值年均增长5.6%。加快财政金融创新，预决算公开、PPP项目模式、政府购买服务等深入推进，交通银行、浦发银行等4家金融机构入驻酒泉，2家农村信用社改制设立为农商银行，小贷公司发展到51家，祁连山药业、华杰电器等企业在“新三板”挂牌。农村土地确权颁证、集体林权综合配套改革、不动产统一登记、疏勒河流域水权改革试点及水价改革深入推进。

　　——过去的五年，我们加快调整产业结构，着力促进转型发展，战略新兴产业规模壮大，现代产业体系逐步形成。大力推进现代农业发展，高效蔬菜、名优果品、现代制种、特色中药材等优势产业面积达到217万亩，建成科技示范园区120个、标准化规模养殖小区982个，培育农业新型经营主体2800多个，建成国家级龙头企业4个、省级龙头企业29个，农业增加值年均增长5.3%，肃州、敦煌建成国家级现代农业示范区。制定实施壮大首位产业、提升传统产业、培育战略新兴产业等行动计划，形成以新能源及新能源装备制造千亿元产业链为主体，煤化工、硅材料、非金属及建材、有色、黑色、石化6个百亿元产业链为支撑的新型工业格局；国家首个千万千瓦级风电基地和首个百万千瓦光电基地建成，风、光电装机分别占全国装机总量的9%和4%，新能源装备制造形成全产业链生产能力。酒泉工业园区升格为国家级经济技术开发区，培育形成中材科技、奥凯种机等7户省级战略性新兴产业骨干企业，全市工业增加值年均增速达到6.8%。深入开展服务业综合改革试点，建成商品交易市场74个，外贸进出口年均增长6.7%，社会消费品零售总额年均增长12.8%。华夏文明传承创新区建设全面推进，文化、体育、健身与旅游业加速融合，敦煌被确定为丝绸之路国际文化博览会永久会址，锁阳城、悬泉置、玉门关申遗成功，敦煌成功创建国家级文化产业示范园区和国家级旅游业改革创新先行区，中国(酒泉)西部户外运动之都建设已具雏形，物流、快递、信息、金融、电商等服务业快速发展，第三产业对经济增长的贡献率达到50%。

　　——过去的五年，我们统筹城乡发展，着力推进城市建设，城乡环境面貌明显改观，城市美誉度和竞争力显著提升。以酒嘉一体化城市规划纲要和酒泉城市总体规划为引领，坚持新区拓展和老城改造并举，完成城市建设投资280亿元，比“十一五”增加2.2倍，七县市区城镇建成区面积达到103平方公里，城镇人均住房面积由24.5平方米增加到27.9平方米，集中供热覆盖面达到90%，建成区绿化覆盖率达到37.4%，城市综合承载能力和带动辐射效应增强。城乡一体化步伐加快，新农村建设取得阶段性成效，农村饮水安全普及率由69%提高到98%，砖混结构住房比重由41.8%提高到62%，楼居式社区化中心村占比达到10%，城镇化水平达到58.3%。生态文明建设稳步推进，森林覆盖率由3%提高到5.5%，单位GDP用水量、单位GDP能耗、污染物排放等约束性指标全面完成，环境质量指数由75.8%提高到85.6%，我市被列为国家级生态文明先行示范区。

　　——过去的五年，我们坚持为民价值取向，着力落实惠民实事，社会保障水平不断提高，社会各项事业全面进步。把改善群众生活、增进民生福祉作为首要任务，五年来用于承办惠民实事和保障民生的财政资金增幅均在15%以上。大力提高就业服务水平，帮助9500名就业困难人员实现就业，累计选拔6398名高校毕业生到基层和企业就业，应届高校毕业生就业率提高到85%。社会保障体系更加完善，全市社会保障综合覆盖率保持在95%以上，企业退休人员养老金、城乡居民基础养老金、城乡居民医疗保险筹资标准及报销水平分别提高50%、25.7%、142%和80%，城乡低保标准分别提高85%和160%，五保集中供养标准提高230%。社会事业全面进步，消除危旧校舍74万平方米，新建改造93万平方米，市级标准化学校创建面达100%，义务教育均衡发展在全省率先通过评估考核，我市被评为全国“两基”工作先进市。研发投入占GDP的比重达2%，科技进步对经济增长的贡献率达到55%。每千人拥有医疗床位5.6张、卫生技术人员6.2人，每千名老人养老床位达到32张，均高于全省平均水平。深化双联行动，认真落实精准脱贫决策部署，结合省上“1+17”工作方案，对接出台84条配套措施，全市移民人均纯收入年均增长20%，五年累计减贫11.8万人，提前实现了整体脱贫目标。

　　——过去的五年，我们坚持转变政府职能，着力加强政府自身建设，行政效能显著提高，建设人民满意政府取得实效。深入开展群众路线教育、“三严三实”“两学一做”专题教育活动，严格执行中央八项规定和省市委“双十条”规定，全面落实“两个责任”，驰而不息整治“四风”，自觉服从市委领导，认真落实市委决定决议，廉洁从政能力和政府行政效能明显提高。积极探索“互联网+制度”的效能监察新模式，围绕中央和省市重大决策执行、重点任务落实、“惠民实事”办理和重大项目推进，全面推行电子政务督察和行政许可一站式服务，认真落实“三重一大”集体决定和主要领导末位表态、不直接分管财务人事工程建设等制度，持续开展工程建设领域招投标、土地及矿产资源招拍挂、政府采购、财政资金使用管理、各项规定落实和干部招录选用等领域常态化监督检查，强化审计监督，大力开展“不作为、慢作为、乱作为”专项整治，下发监察建议书33件次，纠正了一些机关和部门效率低下、推诿扯皮、浪费资源和损害群众利益的问题。

　　各位代表，五年的成绩来之不易。这是省委、省政府和市委坚强领导的结果，是市人大、市政协和社会各界大力支持、有效监督的结果，是全市人民辛勤努力、共同奋斗的结果。在此，我代表市政府，向各位人大代表、政协委员和全市广大干部群众，向各民主党派、工商联、人民团体、离退休老同志，向驻酒人民解放军、武警官兵和公安干警，向中央、省属驻酒单位以及所有关心、支持酒泉发展的各界人士表示衷心的感谢和崇高的敬意!

　　在总结成绩的同时，我们也清醒地认识到，当前政府工作还存在一些不容忽视的问题:一是经济欠发达仍然是基本市情，产业和产品结构不尽合理，加快供给侧结构性改革、转方式调结构的任务依然艰巨；二是基础设施建设滞后，固定资产投资增长乏力，实现可持续发展的任务依然艰巨；三是城乡居民收入持续增长压力较大，巩固脱贫成果、保障改善民生的任务依然艰巨；四是经济外向度低，创业创新内生动力不足，改革开放和创新发展的任务依然艰巨；五是政府效能和依法行政的能力有待提高，加强法治建设、提高履职能力、优化发展环境的任务依然艰巨。对此，我们将高度重视并加以解决。

　　今后五年的目标任务

　　刚刚闭幕的市第四次党代会，在总结过去五年成就、准确把握阶段性特征的基础上，绘就了深入贯彻落实新发展理念，加快建设活力小康生态魅力幸福酒泉的宏伟蓝图。面对新形势、新任务、新要求，我们必须全面贯彻落实市第四次党代会精神，抢抓“一带一路”建设的历史机遇，凝神聚力谋发展，千方百计惠民生，保持经济社会持续健康较快发展。

　　今后五年政府工作的总体要求是:高举中国特色社会主义伟大旗帜，全面贯彻落实党的十八大和十八届三中、四中、五中、六中全会精神，以邓小平理论、“三个代表”重要思想、科学发展观为指导，深入贯彻习近平总书记系列重要讲话和治国理政新理念新思想新战略，按照“五位一体”总体布局和“四个全面”战略布局的要求，牢固树立和贯彻落实创新、协调、绿色、开放、共享发展理念，着力优化经济结构，着力转换发展动能，着力厚植发展优势，着力保障和改善民生，全力谱写活力小康生态魅力幸福酒泉建设新篇章。

　　主要奋斗目标是:提前两年全面建成小康社会，稳步提升小康质量和水平。实现“两个翻番”“五个提升”，经济保持中高速增长，全市生产总值和城乡居民人均可支配收入比2010年翻一番，增速高于全国全省平均水平，固定资产投资、社会消费品零售总额、一般公共预算收入等主要指标增速达到或高于全省平均水平。基础支撑能力、社会文明程度、开放开发水平、人民群众获得感和党的建设科学化水平显著提升。

　　——坚持在发展动能转换中提质量、增效益，着力推进活力酒泉建设。把改革创新放在发展全局的核心位置，加快构建以改革创新为发展动能的经济体系和发展模式。认真研究落实中央、省市委各项决策部署和政策举措，坚持正确方向、问题导向、为民取向、破题指向，扎实推进供给侧结构性改革。深化行政体制、财税体制、投融资体制等重点领域和关键环节改革，更好地发挥政府的引导作用和市场在资源配置中的决定性作用。突出企业在科技创新中的主体作用，通过政策支持、项目扶持、融资倾斜、资金奖补等措施，鼓励大禹节水、奥凯种机等一批重点科技型企业加快自主创新，支持金风科技、酒泉正泰等重点企业与科研院所加强关键技术产学研协同创新，加速科技成果转化，提高矿产采选、农业机械、农产品加工、石油化工、建筑建材、金属冶炼及压延加工、印刷包装等产业装备水平，促进资源精深加工和深度转化，扶持小微企业上层次、上规模，着力提高经济运行质量和效益。推进“智慧城市”建设，搭建“互联网+”运行平台，为众创、众包、众扶、众筹提供支撑服务。挖掘驻地部队和企业高端人才汇聚的优势，完善激励各类人才创业创新的政策措施，建立健全容错纠错机制，强化诚信体系建设，全力营造干事创业的宽松环境。

　　——坚持在提升发展协调性上补短板、强产业，着力推进小康酒泉建设。大力推进区域统筹、产城融合、城乡互动，在民主法治、资源环境、科技投入等短板上下功夫，着力提高文化产业占比，努力缩小城乡收入差距，提前两年在全省率先全面建成小康社会，到2020年建成更高水平、更高质量、人民群众获得感显著提升的全面小康社会。培育云计算、大数据等高耗能产业，加快发展新材料、新型煤化工、高端装备制造等新兴产业，推进风光储输一体化、资源综合利用、清洁电能消纳、产业链配套延伸项目发展，促进产业迈向中高端，增强现代工业支撑能力。提升肃州、敦煌国家现代农业示范区标准化建设水平，发挥示范引领推广作用，做精做优特色优势产业，大力推广先进设施农业、节水灌溉技术和种养加标准化体系建设，提高品牌效应和规模效应，增强农业基础保障能力。发挥历史底蕴深厚、文化遗存众多、民俗风情浓郁的资源优势，深入推进“文化+”发展战略，开发独具特色的旅游产品，着力打造国际旅游目的地和区域旅游集散地，把文化旅游体育产业培育成战略性支柱产业。优化提升商贸、会展等传统服务业，培育壮大金融服务、现代物流、电子商务和信息等现代服务业，加快发展商务服务、科技服务、健康养老等新型服务业，重振丝路重镇雄风。

　　——坚持在建设美丽宜居酒泉上求自然、重保护，着力推进生态酒泉建设。“酒泉酒美泉香，雪山雪白山苍”，空气、阳光、水质是酒泉最宝贵的财富，最具优势的核心竞争力，必须坚持“生态立市”战略不动摇，划定并坚守生态底线，组织实施“三北”防护林、退耕还林还草、敦煌水资源综合利用与生态保护、党河中上游生态恢复治理、引哈济党、祁连山生态保护与综合治理等一批重大项目，严格落实地下水超采区禁采措施，全力构建“一区四屏”为主体的区域生态格局，打造河西祁连山内陆河生态安全屏障。推动绿色循环低碳发展，用新《环保法》规范发展行为，促进传统产业改造提升，巩固绿色产业和循环经济发展成果，工业固体废物和农作物废弃物综合利用率达50%以上。全面落实大气、水、土壤污染防治计划，推广清洁能源供暖、清洁水源管控、污水综合治理，确保大气优良天数逐年提高，人均公共绿地面积达到15平方米以上。强化规划控制，严格规划执行，倡导绿色生活方式，提高城市管理水平，创造生态优美、生活舒适的良好环境。

　　——坚持在打造对外开放格局上高站位、搭平台，着力推进魅力酒泉建设。主动融入“一带一路”国家战略，深化酒泉特有资源的认识，拓宽全球视野，打造国际品牌。充分挖掘丝绸之路(敦煌)国际文化博览会的深刻内涵，加强与各个国家、城市在文化旅游产品研发生产、文化艺术创作展示等方面的交流合作和民间往来，促进各个国家、各个民族的文化交流与合作，加强文化贸易、创意旅游等共同合作发展。积极扩大国际新能源博览会品牌影响，依托酒泉国家级经济技术开发区、新能源产业示范基地，放大能源建设、装备制造、检验检测、产品研发、备品备件交易优势，加强对外合作交流，带动新能源产能、技术和标准输出，实现产业转型升级和可持续发展。着力增强国家现代农业示范园区辐射效应，加大科技投入，培育优良种质资源，规范水肥使用标准和现代高效节水农业模式，发展种子产业园、水产品交易中心、清真牛羊肉精深加工等项目，提升农业和农产品服务水平。立足通道优势和地处四个省会城市中间节点的优势，规划布局西北物流基地、建筑建材产业园、中东部商品交易及新能源装备集散等功能完善的物流园区，培育大数据、“互联网+”、物联网等线上线下新兴业态，积极引进京东等电商巨头落户酒泉，建设中国西部商贸物流枢纽城市。顺应新时期健康消费新趋势，深度挖掘多样性地貌和多元化气候资源，积极推进以户外运动、特色赛事和休闲养生为主体的文化旅游体育产业融合升级，积极培育“走、跑、飞、驾、攀、骑、滑、蹦”八大户外业态，真正把酒泉打造成为国际知名的中国西部户外运动之都。

　　——坚持在保障和改善民生上办实事、增福祉，着力推进幸福酒泉建设。以人为本，发展成果让全市人民共同享受，是我们永恒不变的追求。要通过政策激励、困难援助、培训提升、输转促进和公共就业服务等措施，将城镇登记失业率控制在3.5%以内。持续提高城乡低保、医保、大病救助、残困帮扶标准，完善健康养老服务体系，让身处困境者得到更多关爱。以棚户区改造为重点，提高住房保障水平，确保城镇常住人口住房保障覆盖率达到20%。完善收入分配调节政策措施，多渠道增加城乡居民经营性、工资性、转移性和财产性收入，城乡居民人均可支配收入年均增长8%以上。加快城乡教育一体化发展，优化公共教育资源配置，提升城乡教育均等化水平。深入推进医疗卫生体制改革，提高医疗卫生服务保障水平，切实解决群众看病难、看病贵问题。加快公共文化基础设施建设，完善现代公共文化服务体系，推动基本公共文化服务标准化、均等化发展，满足人民群众不断提升的精神文化追求和享受。落实食品药品安全监管制度，健全安全生产监管责任体系，保障人民群众健康安全。加快民族地区发展，持续开展民族团结进步教育创建活动。深化社会治理创新，健全治安防控网络体系，不断提高人民群众安全感。努力使今后五年成为全市人民幸福指数明显提高、得到实惠更多的五年。

　　2017年工作建议

　　2017年，是贯彻落实市第四次党代会精神的第一年，也是深入实施“十三五”规划的重要一年。做好明年的政府工作，对建设活力小康生态魅力幸福酒泉具有十分重要的意义。

　　全市经济社会发展的预期目标是:地区生产总值增长7.5%左右，固定资产投资增长10%左右，社会消费品零售总额增长9%左右，公共财政预算收入增长8%左右，城镇居民人均可支配收入增长8%左右，农村居民人均可支配收入增长9%左右，单位生产总值能耗降低2%左右，主要污染物排放控制在省定目标以内。重点抓好七个方面的工作:

　　一、扩大有效投资，增强经济发展后劲。加快建设政府性投资项目，强化市场性投资项目服务，开工建设重点项目1380个，完成固定资产投资1336亿元。紧扣国家政策走向，谋划争取一批符合产业政策、支撑长远发展的重大项目。全力抓好敦当高速、瓜敦高速、国道312线改造等项目，加快推进肃航公路、马鬃山口岸公路等工程，积极做好酒金公路、肃北至德令哈公路、阿克塞至若羌公路、酒额铁路、敦煌客运专线等项目前期工作。争取启动引哈济党、洪水河水库、马鬃山引水等水利工程，加快河西走廊国家级节水示范区建设。持续抓好敦煌水资源综合利用与生态保护、北大河生态综合治理、祁连山水源涵养区、酒泉生态文明示范区等项目建设。采取“走出去”“请进来”和组团招商、委托招商、以商招商等方式，聚焦长三角、珠三角、3个“500强”和行业龙头企业推介招商，注重引进效益高、带动强、成长性好、财税贡献大的产业发展项目。推出和落实一批PPP项目，放宽准入条件，引导社会资本投向市政、交通、生态等公共服务领域。

　　二、狠抓重点改革，释放创新发展活力。坚持问题导向，从重要领域、重点环节发力，着力抓好各项改革任务。继续简政放权、简化程序，严格落实“三张清单一张网”，推进投资审批、商事制度、事业单位改革，加快相对集中行政审批权和处罚权改革，加强事中事后监管，用政府权力减法换来市场活力乘法。进一步完善市、区和经开区财政管理等体制机制，科学合理划分事权和支出责任。完善预算管理体系，清理规范重点支出同财政收支规模挂钩事项。规范政府性债务，健全政府投资项目资金统筹平衡和债务风险预警防范机制。加强国有资产管理，全面清收闲置资产，实现国有资产保值增值。加强土地节约集约利用，完善不动产统一登记制度。实施设施农业、水面渔业、休闲农业、国有林场等产权改革，全面完成农村承包地经营权确权登记颁证，鼓励农户依法自愿有偿流转承包地，探索开展土地股份合作、联合或土地托管试点，深入推进水权确权、水价综合改革。抓好瓜州资源综合利用产业园区售电侧改革试点，扩大电力直接交易覆盖面，完善供水、供热、污水处理等价格体系，继续清理经营性收费和中介收费，促进市场要素合理配置。

　　三、转变发展方式，推动农业提质增效。加快发展现代农业，力争农业增加值增长5.5%。壮大多元经营主体，扶持培育种养大户、家庭农场、农民专业合作社等新型主体，发展多种形式的规模经营。紧盯市场需求，大力发展蔬菜、名优果品、现代制种、特色中药材、草食畜等优势特色产业集群，优势特色产业面积增长3%，新增肉羊饲养量30万只。促进农业与乡村旅游、健康养老深度融合，培育农村电商、订单农业等新业态，拓展农业发展新空间。加快农村社会化服务体系建设，加强农业科技创新。实施化肥农药零增长行动，培育农产品知名品牌和商标，健全农产品质量全过程监管追溯体系和现代农业推广服务体系。扶持种业发展和交易市场建设，形成聚集和扩散功能。深化精准扶贫精准脱贫，深入推进富民增收产业培育行动，进一步改善移民区基础设施和基本公共服务水平，不断巩固提升脱贫成效，确保脱贫人口人均收入增幅高于全市平均水平。

　　四、优化结构布局，力促工业转型升级。着眼建设“169+1”现代产业体系，加快供给侧结构性改革步伐，深入推进产业转型升级，改造提升传统产业，培育壮大战略性新兴产业，力争工业增加值增长6%。建成酒泉至湖南±800千伏特高压直流输电工程，全面实施500万千瓦风电、1350兆瓦光电项目，完成常乐电厂400万千瓦调峰火电项目年度建设任务，开工建设55万千瓦光热发电示范项目。坚持以资源开发推动装备制造发展，重点引进关键零部件、光热发电等补链延链项目，开展“科技入园”行动，提高新能源装备制造技术创新水平，组建行业协会和产业联盟，建成备品备件购销体系，促进装备制造业优化升级。加快酒泉核技术产业园、中以(酒泉)中小企业国际节水产业合作园区建设，大力发展煤化工、硅材料及制造、非金属采选加工及建材等循环经济产业链，加快发展石材精深加工，着力化解过剩产能，淘汰低端落后工艺，提高资源综合利用水平。加快酒泉绿色大数据中心和产业园建设，积极推进电子政务、城市管理等智慧项目建设，构建畅通、便捷、高效的信息化社会。

　　五、发挥平台效应，提升三产发展水平。着力打造人文交流高地、商贸物流枢纽、户外运动之都和休闲养生福地，促进生产性服务业向专业化和价值链高端延伸，生活性服务业向精细化和高品质转变，第三产业增加值增长9%。深入实施“文化+”发展战略，持续放大丝绸之路(敦煌)国际文化博览会效应，大力发展文化会展、演艺娱乐、创意设计、健身休闲等产业，文化产业增加值增长20%以上。加快肃州湿地公园、金塔恐龙地质公园、玉门地质公园、肃北盐池湾冰川、阿克塞沙漠公园等旅游项目建设，着力建设以东三县为主的区域旅游集散地和以西四县为主的国际旅游目的地，力争旅游接待人数、旅游收入均增长20%以上。积极创建国家智慧物流配送示范基地，加快推进丝绸之路国际商贸城、万达广场等大型商贸设施、商业中心和特色商业街建设。加快兴业银行、浙商银行入驻步伐，支持西域新材等企业上市融资，力争直接融资增长10%以上。

　　六、强化统筹协调，推进城乡一体建设。深化“多规合一”工作，修编城市总规，科学编制综合管廊专项规划、城市交通专项规划、海绵城市规划，修编北大河两岸、高铁片区、汉唐文化新区等区域控制性详规，为彰显城市魅力提供引领和支撑。坚持新城开发与老城改造并举，加快城乡道路、供水供热供气、污水处理等基础设施配套完善和改造维护力度，切实增强承载服务功能。加大重点区域环境治理，认真解决交通峰值拥堵、车辆乱停乱放、商户占道经营等问题。着力补齐环境短板，加大污染防治力度，打好大气、水体、土壤污染防治攻坚战，建成并达标运行第二污水处理厂，完成酒泉城区第三水厂水源调整和第二水厂治理项目。认真做好新型城镇化试点验收工作，抓好肃州区国家中小城市综合改革试点和敦煌市国家新型城镇化综合改革试点。积极落实居住证制度，加快农业转移人口市民化，推进城镇基本公共服务常住人口全覆盖，城镇化率提高1.6个百分点。积极开展美丽乡村、美丽宜居小镇、村庄示范创建活动，抓好酒泉玉文化特色小镇建设，打造一批特色鲜明、功能完善、环境优美的宜居村镇。稳步推进城镇保障性安居工程建设，因地制宜化解房地产库存，促进房地产市场健康发展。

　　七、保障改善民生，增进人民群众福祉。认真落实就业扶持政策，加强就业技能培训，不断提高就业竞争能力和创业能力，城镇登记失业率控制在4%以内。统筹推进社会保障体系建设，全面实施全民参保登记计划，城乡居民基本养老和城镇居民基本医疗保险参保率巩固在98%以上，实现各项社会保险制度对法定人群全覆盖。继续提高企业离退休人员和城乡居民基本养老金、伤残津贴、失业金等社会保障待遇，加快机关事业单位养老保险制度并轨改革。提升教育公平度和满意率，引导学前教育普惠化、特色化发展，实现行政村幼儿园全覆盖，统筹城乡教育资源，切实缓解城区就学压力。深化健康促进模式改革、公立医院综合改革和医疗服务价格改革，整合城乡居民基本医疗保险，落实分级诊疗和医师多点执业。进一步完善基层医疗卫生机构运行机制，提升基层医疗服务能力。加强传染病防控能力建设，严防人间鼠疫疫情发生。实施基层中医药服务提升工程，扶持中药产业发展。认真落实全面两孩政策，促进人口长期均衡发展。深入推进平安酒泉建设，完善社会治安防控、公共安全管理和应急抢险处置体系，严厉打击各类违法犯罪行为。完善基层食药监管体制，强化安全生产责任体系建设，建立健全安全生产诚信体系和“黑名单”制度，保持打非治违高压态势，坚决遏制重特大事故发生。加强多元化纠纷解决体系建设，有效解决信访问题、化解社会矛盾。进一步加强双拥工作，推动军民深度融合发展。继续做好统计、人防、边防、地震、气象、妇女儿童、民族、宗教、残疾人、台侨、档案、史志、外事、驻外联络等工作。

　　切实解决民生问题，集中力量办好十件惠民实事:一是推荐1000名以上高校毕业生进企业见习就业；二是开展农民工职业技能培训1.5万人以上；三是免费办理困难群众法律援助案件2800件以上；四是60岁以上城乡残疾老人、困难老人生活补助标准提高100元以上，80岁以上高龄老人生活补助标准提高1倍；五是建成酒泉第七中学；六是取消市级公立医院药品加成，减轻群众看病负担；七是建成酒泉市人防训练基地和体育馆；八是实施全市自备水源井关停并网工程，解决市民安全饮水隐患；九是完成村村通宽带网络工程，实现全光网城市目标，新建100个农村电商服务站点；十是新改建农村公路600公里。

　　深入推进政府自身建设

　　新常态、新理念、新目标、新任务，对政府工作提出了新的更高要求，我们将认真践行全心全意为人民服务根本宗旨，坚决贯彻落实市委决策部署，进一步加强自身建设，切实改进工作作风，为开创政府工作新局面提供坚强的组织和队伍保障。

　　一是加强理论武装，建设学习型政府。认真学习贯彻习近平总书记系列重要讲话精神和治国理政新理念新思想新战略，深化“三严三实”专题教育，纵深推进“两学一做”学习教育，增强党性修养，强化政治意识、大局意识、核心意识、看齐意识，确保中央和省、市委重大决策部署全面贯彻落实。加强对国家宏观政策、产业政策和微观经济的学习研究，深化对本地区本行业发展瓶颈和关键环节的准确把握，提高科学执政、民主执政的能力和水平。

　　二是转变政府职能，建设服务型政府。坚持把服务人民、服务发展作为根本目的，遵循市场发展规律，健全政府购买公共服务机制，积极推进公共服务职能向社会组织转移，引导社会资本参与公益性事业投资运营。全面推行政务公开，加强政务平台和政府网站信息共享建设，扩大信息公开范围，畅通政府与公众互动渠道，完善服务群众快速响应机制。加强管理创新和制度创新，破除制约发展的思想认识和制度障碍，综合运用现代科技手段提高办事效率，增强政府执行力和公信力。加强社会综治、市场监管、综合执法、便民服务等平台建设，着力构建统一高效的社会治理体系。

　　三是严格依法行政，建设法治型政府。牢固树立社会主义法治理念，增强运用法治思维和法治方式解决问题、化解矛盾的能力。坚持法定职责必须为、法无授权不可为，严格按照法律规定的权限和程序行使权力、履行职责。完善重大事项决策制度，严格执行决策法定程序，完善终身责任追究及责任倒查机制。认真落实行政执法责任制，全面梳理政府权力事项，规范执法行为，完善执法程序，推动执法资源统筹利用、合理配置。自觉接受人大及其常委会的法律监督、工作监督和政协民主监督，认真办理人大代表建议和政协委员提案，虚心接受社会公众监督，切实保障广大群众的知情权、参与权、表达权和监督权。

　　四是狠抓反腐倡廉，建设廉洁型政府。认真落实党风廉政建设责任制，强化两个责任体系，严格执行党内政治生活《若干准则》和《党内监督条例》，做模范遵守党章党纪的表率。强化重点领域制度建设，加强行政监察和审计监督，加大对项目招投标、征地拆迁、民生项目和政府性工程建设等重点领域的监管力度，扎实开展对公共资金、国有资产、国有资源和领导干部经济责任审计，健全审计整改机制，推进审计监督全覆盖，促进行政权力规范公开公平行使。认真落实省、市委“双十条”规定和廉洁自律各项要求，坚决查处部门和行业不正之风，决不允许欺压群众、损害和侵占群众利益，坚决反对形式主义、官僚主义、享乐主义和奢靡之风，着力营造风清气正的良好环境。

　　各位代表，蓝图催人奋进，圆梦需要实干。让我们更加紧密地团结在以习近平同志为核心的党中央周围，在省委、省政府和市委的坚强领导下，紧紧依靠全市人民，解放思想、脚踏实地、开拓进取，为建设活力小康生态魅力幸福酒泉而努力奋斗!
